# Laser Operating Windows Prediction in Selective Laser-Melting Processing of Metallic Powders: Development and Validation of a Computational Fluid Dynamics-Based Model

**DOI:** 10.3390/ma13061424

**Published:** 2020-03-20

**Authors:** Maria Rita Ridolfi, Paolo Folgarait, Andrea Di Schino

**Affiliations:** 1 Seamthesis Srl, Via IV Novembre 156, 29122 Piacenza, Italy; mariarita.ridolfi@seamthesis.com (M.R.R.); paolo.folgarait@seamthesis.com (P.F.); 2 Dipartimento di Ingegneria, Università degli Studi di Perugia, via G. Duranti, 06125 Perugia, Italy

**Keywords:** additive manufacturing, selective laser melting, numerical model, laser operating window, metallic alloys

## Abstract

The rapidly ascending trend of additive manufacturing techniques requires a tailoring of existing solidification models and the development of new numerical tools. User-friendly numerical models can be a valid aid in order to optimize operating parameter ranges with the scope to extend the modelling tools to already existing or innovative alloys. In this paper a modelling approach is described simulating the generation of single tracks on a powder bed system in a selective laser melting process. The approach we report attains track geometry as a function of: alloy thermo-physical properties, laser speed and power, powder bed thickness. Aim of the research is to generate a numerical tool able to predict laser power and speed ranges in manufacturing porosity-free printed parts without lack of fusion and keyhole pores. The approach is based on a simplified description of the physical aspects. Main simplifications concern: the laser energy input, the formation of the pool cavity, and the powder bed thermo-physical properties. The model has been adjusted based on literature data providing the track’s geometry (width and depth) and relative density. Such data refer to different alloys. In particular, Ti6Al4V, Inconel625, Al7050, 316L and pure copper are considered. We show that the printing process presents features common to all alloys. This allows the model to predict the printing behavior of an alloy from its physical properties, avoiding the need to perform specific experimental activities.

## 1. Introduction

Selective laser melting (SLM) is one of the most widely spread and successful powder bed fusion-based additive manufacturing technologies. In SLM, melting and solidification of a small volume of powder is achieved using a high-intensity laser scanning over a layer of powder. Finally, the part is obtained connecting partially overlapping tracks consolidated over any single layer, with traces previously scanned on few layers below, partially re-melted and consolidated. 

The main concern deriving from this technique is attaining a fully dense part out of this interconnection of tracks. All the expected mechanical properties of an additive manufactured part, such as strength, ductility, creep and fatigue behaviors, largely, although not uniquely, depend on the presence of porosities. The formation of defects of additive manufacturing, including porosity, is the object of extensive study through the modeling at the micro and macro scale applied to laser powder bed fusion process [1], and to solid freeform fabrication [2]; and through deep experimental analyses carried on commercial alloys such as AlSi10Mg [3], Ti6Al4V [3,4], and 316 L stainless steel [5].

Some alloys show a higher natural tendency to porosity formation. Aluminum- and copper-based alloys require high laser-specific energy to counterbalance their high reflectivity and thermal diffusivity making them particularly prone to porosities caused by lack of fusion. Extensive studies investigated windows of parameters required to produce high-density parts from aluminum alloys [6,7,8]. Pure copper density has been also investigated by using high-power laser and selective electron beam melting techniques [9,10]. 

Moreover, printed parts of some widely studied alloys, like stainless steel 316 L, induced deeper investigations to find optimal printing strategies in the aim of increasing the build rate, while maintaining low porosity levels [11,12].

The right choice of process parameters is of fundamental importance to obtain a porosity-free component, and should be based on powder composition and size distribution [13,14]. Theoretically, the process parameter list comprises all the following: layer thickness, hatch, laser spot diameter, scanning speed, and laser power. Layer thickness comes from considerations about the resolution of the part details and on the target surface finish, while laser spot diameter is often fixed on commercial machines. The optimal process design relies eventually on the right choice of laser power and velocity as well as on hatch distance. Hence, in view of optimizing the SLM process for producing dense part of a given metal alloy, one should have tools to define the operating window in the P-v (laser beam Power–velocity) space, depending on metal alloy composition and powder granulometry.

A way to define the printing operating window consists in printing and observing samples, according to a test matrix of various power and velocity combinations. Alternatively, the same matrix can be produced by numerically simulating the process, minimizing the experimental work and consequently time and costs. The welding process at the scale of the bead is numerically modelled with simplified approaches solving the purely thermal problem using the finite element or finite volume techniques with the aim of shortening the computation time [15,16]. The same modeling approach is applied in the full-scale layer-wise AM model developed by AlMangour et al. [17] for the study of the printing process of TiC-reinforced 316L stainless steel. The solution of thermo-mechanical problem induced, moreover, the development of numerical tools of practical application, in order calculate process-induced stresses and strains based on the detailed temperature field [16], also taking into account the influences of the microstructure transformations [18]. 

More sophisticated multi-physical approaches intimately describe and give insights on complex phenomena underlying the mechanisms of powder bed melting, solidification and consolidation in SLM, at the scale of the single powder particles [19,20]. The mechanism of formation of keyhole porosities has been also visualized through thermo-fluid dynamic modeling [21].

The scope of the work described in this paper is to create a modelling tool for generating processing maps of metal alloys applicable to the laser powder bed melting technology. The processing windows obtained must be based on reliable predictions of melted volumes and density of interconnected tracks. The model has been developed with the basic idea of adequately representing the process within a simplified approach in order to reduce the computational time for practical use. For this reason, the model contains net simplifications of the complex reality (surface tension effects, evaporation and plasma formation are not simulated), aiming for a simplified simulation of heat transfer, leading to a reliable prediction of shape and dimensions of the melted volume. Based on these premises, the model assumes a semi-empirical nature since it uses parameters whose dependence on alloys properties and input energy is found fitting the model results with experimental data.

Furthermore, using such a model should avoid resorting to experimental testing to be carried on each alloy object of analysis. 

To achieve the target, a proper strategy of calibration has been conceived. After appropriate validation and adjustment, the model is to be used to span over the process parameter ranges allowed by the specific additive manufacturing (AM) machine. The model results provide laser operating windows for alloys of any composition, representing the fields of operating parameters for which both lack of fusion and keyhole porosity are avoided. This implies as well the need to calculate relative density of macroscopic printed part, resulting from single tracks interconnections, evaluated out of the geometry of the modelled single track.

At the present stage, the model has been validated and calibrated using literature experimental findings from researches performed on some alloys having different base metals. These reference works aimed at understanding the printing performance at varying laser parameters within large ranges. Observations of track microstructure and geometry, density of printed samples, measures of effective laser absorptivity have been withdrawn from these literature sources.

The printing process consists in a complex phenomenon interrelation. The powder bed surface in contact with the ambient atmosphere is basically exposed to the laser irradiation and partly reflects the incident energy, according to its reflectivity; the remaining energy is diffused inside the material. 

The surface tension gradient provokes Marangoni convection [22,23]. At printing regime conditions, most of the exposed surface is the liquid pool-gas interface, holding almost flat until the laser energy becomes high enough to cause intense evaporation and boiling. At the onset of metal boiling, an increasingly deeper cavity forms, increasingly entering laser-specific energy. As the cavity deepens, much energy is entrapped due to multiple ray reflections against the cavity interface [24,25]. Due to this mechanism, a shallow cavity intercepts less energy than a deep keyhole cavity. This results in a continuous increase of the effective laser absorbance reaching the maximum close to unity, when the keyhole is fully developed. The minimum laser energy is absorbed when the pool surface is almost flat, i.e., in the conduction mode. The absorptivity in this condition is similar to the natural absorptivity of the metal surface.

In conclusion, raising the laser specific energy makes the melt pool geometry transforming from wide and flat into narrow and deep.

## 2. Materials and Methods

The complex physical scheme is modelled taking into consideration the restrictions dictated by the need to shorten the computing time. For this reason, surface tension forces and Marangoni effect, boiling, recoil pressure and cavity formation are not simulated. Nonetheless, the consequences of the cavity formation are taken into account in the heat-transfer analysis as: (1) increased effective laser energy absorption caused by multiple laser rays’ reflections when the cavity forms; (2) increased depth of laser energy penetration following the cavity deepening. Laser irradiation is simplified into energy per unit volume applied as boundary condition as explained further on.

The model adopts the finite volume technique to perform transient thermo-fluid-dynamics analyses of the printing process, and is developed using the commercial code ANSYS^®^ Fluent, Release 17.1, developed by ANSYS, Inc. (Canonsburg, PA, USA). This choice offers the possibility of further model integrations with surface tension, pool convection and gas-cooling effects.

The model is based on the solution of the transport equation applied to mass and momentum:

(1) $$\frac{\partial \rho }{\partial t}+\nabla \left(\varrho \vec{v}\right)=0$$

(2) $$\;\rho \frac{\partial u_{i}}{\partial t}+\rho \frac{\partial \left(u_{i}u_{j}\right)}{\partial x_{j}}=\frac{\partial }{\partial x_{i}}\left[\mu \left(\frac{\partial u_{i}}{\partial x_{j}}+\frac{\partial u_{j}}{\partial x_{i}}\right)\right]-\frac{\mu }{K}\left(u_{i}-u_{i}^{s}\right)-\frac{\partial p}{\partial x_{i}}+\rho g_{i}\;$$

and to energy:

(3) $$\frac{\partial }{\partial t}\left(\varrho H\right)+\nabla \left(\varrho \vec{u\;}H\right)=\nabla \left(k\nabla T\right)+S_{v}$$

The second term of the right-hand side of Equation (2) accounts for Darcy’s damping force due to the flow of liquid metal through the mushy zone represented as a porous medium of permeability K, obtained with the Karman–Kozeny model [26], which for the dendritic arrangement in the mushy zone assumes the form [27]:

(4) $$K=\frac{PDAS^{2}}{180}\frac{f_{l}^{3}}{\left(1-f_{l}^{2}\right)+0.001}$$

expressed in terms of the primary arm spacing (PDAS) and of the liquid fraction (*f_l_*). The effect of adding this term is negligible for most metal alloys, being the mushy zone extension very small at such high cooling rates. Otherwise, the fluid-dynamics through the mushy zone could affect the thermal field when processing alloys have large solidification intervals.

**Figure 1:**
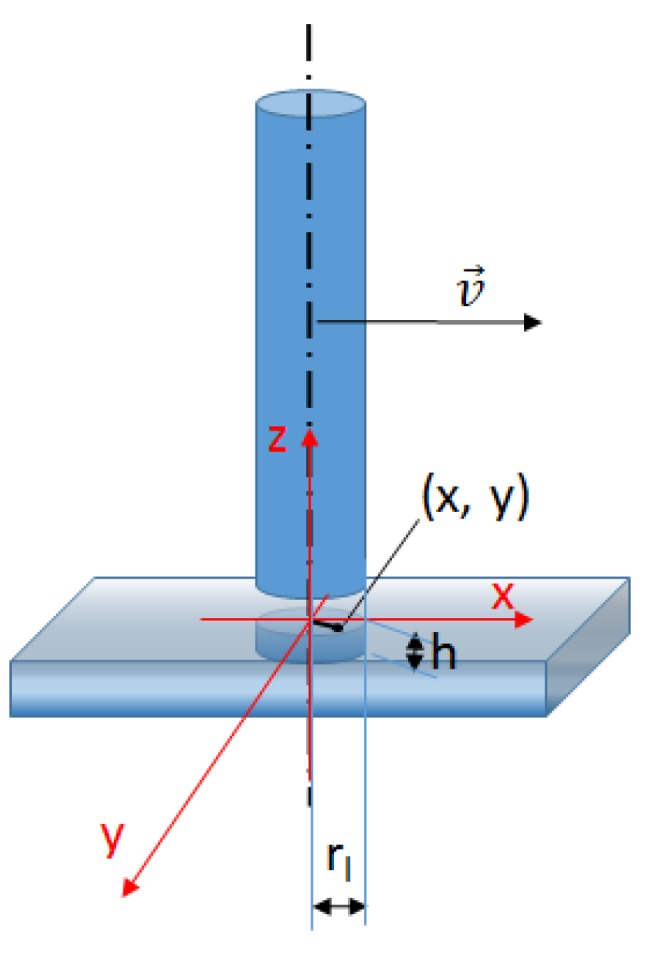
Scheme showing the ideal cylindrical volume inside which the laser energy source is applied, moving with the laser beam at velocity *v*.

The energy input coming from the laser beam is given in the last term of the right-hand side of Equation (3) in terms of energy per unit volume, following the approach reported in [22]. The method consists in applying the heat input, at each time step of the transient analysis, to cells enclosed in a virtual cylindrical volume located just below the laser spot. The scheme in Figure 1 shows the moving laser beam and the volume where energy is applied. *S_v_* assumes the following expression:

(5) $$S_{v}=\frac{D\alpha \eta P}{\pi r_{l}^{2}h}e^{-D\frac{x^{2}+y^{2}}{r_{l}^{2}}}$$

with *α* laser absorptivity and *η* laser efficiency. *α* is the fraction of incident laser power, absorbed beneath the powder bed–gas interface, while *η* the fraction of nominal laser power effectively reaching the interface. A constant value of 0.85 has been attributed to *η* in all the performed analyses. *h* and *α* have been used as calibration parameters for each simulation to fit the measured track geometry.

Temperature dependency has been attributed to thermal conductivity and specific heat of the simulated alloys in the range between room temperature and liquidus temperature. Conditions of incipient cavity drilling are supposed to meet conditions of liquid metal boiling initiation [23,28]. This occurrence is used in the model calibration. The printing process is simulated for energy input experimentally marking the threshold from conduction to vaporization/cavity formation. In this process, the liquid metal boiling is expected as maximum temperature inside the pool. The effective thermal conductivity in the liquid pool has been used as a calibration parameter. It is varied until reaching good fitting between the calculated maximum pool temperature and the boiling temperature. Results on analyses performed over different alloys show that the real liquid thermal conductivity must be increased to obtain good fitting. This could make sense as the artificially increased value of liquid thermal conductivity accounts for effects induced by intense convection, Marangoni flow, etc., not well described in this simplified model. Process conditions marking the transition from conduction to vaporization are detected in the experiments by the onset of a steep rise of track dimensions [29] and effective laser absorptivity [24].

The powder bed is simulated as a continuum having density and specific heat calculated as volume fraction-weighted average of gas and metal respective properties. Thermal conductivity has given a value lower than what would result from the volume fraction-weighted average. A simplified scheme of heat transfer has been introduced, which will be object of further study and refinement.

**Figure 2:**
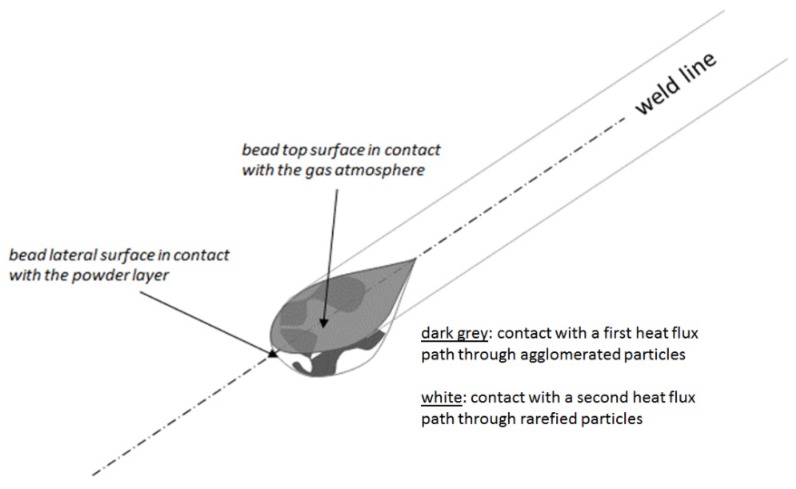
Scheme showing the bead surface in contact with the powder layer and its subdivision into regions contacting powder–gas mixture zones having different density.

According to the scheme shown in Figure 2, heat is considered flowing out of the melt pool through two different parallel paths. The first, corresponding to a given fraction of the melt pool surface (*β*), is formed by powder particles contacting each other. The second, corresponding to the remaining (1-*β*) fraction of the melt pool surface, is formed by looser powder particles. Denoted as *f_min_* and *f_max_* the gas fraction respectively in the first and second path, the effective powder conductivity is calculated according to Equation (6):

(6) $$k=\beta k_{1}+\left(1-\beta \right)k_{2}$$

*k*_1_ and *k*_2_ being the conductivities along each of the two paths. They are obtained by the inverse of the series of thermal resistances of metal and gas:

(7) $$k_{1}=\frac{k_{gas}k_{metal}}{f_{min}k_{metal}+\left(1-f_{min}\right)k_{gas}}$$

(8) $$k_{2}=\frac{k_{gas}k_{metal}}{f_{max}k_{metal}+\left(1-f_{max}\right)k_{gas}}$$

At the present stage, evaluations of the effective conductivity have been made for different sets of *β*, *f_min_* and *f_max_* values, consistent with the powder bed gas fraction. The resulting effective thermal conductivity is about 12%–14% of the metal conductivity, depending on the gas composition, imposing 0.4 as the gas fraction in the powder bed. This approach is designed to allow for future analysis of effects due to powder rarefactions or agglomerations, provided its specific calibration.

The simulation domain consists of a parallelepiped including the platform, one powder layer and the gas atmosphere. Only one half of the track is simulated for symmetry reasons, cut with a vertical longitudinal plane. All the other boundaries are given adiabatic conditions and zero velocity, once the domain dimensions have been properly set largely exceeding the volume where the laser energy is dissipated for all the analyzed process conditions. A multiphase fluid-dynamic analysis is performed using the volume of fluid technique (VOF), with two phases representing gas and metal, excluding surface tension modelling. Heat transfer between the two phases accounts for conduction and natural convection through gas and liquid metal. Radiation is also simulated using the P-1 model implemented in ANSYS Fluent. Sensitivity analyses on the cell size and total transient time of simulation have been performed to ensure that the results do not vary with further decreasing the cell size and that regime temperature field is attained. Mesh adapting has been applied, based on local temperature, in order to refine only the hotter region of the domain. Finally, cell size as small as 3 μm and 1 μs time step have been employed. The transient time of simulation has been assessed to provide a 1 mm long track, depending on the scanning speed.

The calibration analysis has been carried out on five alloys for which measures of track dimensions, part relative density, and total absorptivity are available in the literature [15,24,29,30,31].

Simulations have been performed using process conditions of the experiments, scanning the operating ranges from lowest to highest laser-specific energies (P/v). At low energy input, the values of absorptivity (*α*) and depth of penetration of the laser energy (h) are those corresponding to the conduction mode and they are referred to as *α*_0_ and h_0_. These two values have been used as calibration parameters for all the analyzed alloys. At the end of the calibration it was concluded that good fitting is achieved by setting:

1. *α*_0_ equal to the absorptivity of the flat metal surface, as derived from literature data of reflectivity at infrared wavelength [32], also taking into account reflectivity dependence on temperature [33];
2. *h*_0_ equal to the powder layer thickness plus 10 microns, which could be consistent with the high permeability of the powder bed to laser rays, due to its scarce compactness.

All the experimental analyses reported in [15,24,29,30] refer to single tracks printed on a single powder bed lying on a platform made of the same alloy. Accordingly, the model simulates only one powder layer of nominal thickness. Results in terms of density of printed cubic specimens realized with different laser parameters and powder layer thickness, are given in [30,31]. To simulate these experiments the effective layer thickness has been simulated. All the experiments have been performed with laser systems with 1.06–1.07 μm wavelength. Measures of width and depth are provided in [24,29,30]. Only measures of track cross section area are available in [15]. In addition, also effective absorptivity results, obtained through direct calorimetric measurements are found in [24].

Table 1 shows the tested alloys along with their relevant thermo-physical properties and corresponding literature sources of measures used in the model adjustment. Thermo-physical properties of pure copper have been derived from [34], reflectivity indexes from [32,33]. All other data comes from previous studies performed on alloys’ thermo-physical properties carried out using JMatPro^®^, Release 4.0, developed by Sente Software Ltd. (Surrey, UK).

## 3. Results

**Figure 3:**
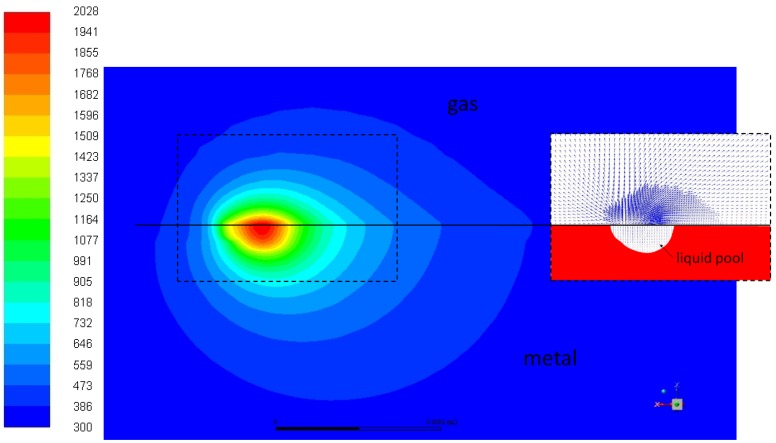
Thermal field and detail of fluid-dynamics around the melt pool, on the longitudinal symmetry plane (case of pure copper printed at 800 W and 1000 mm/s).

Figure 3 shows, as examples, thermal and fluid-dynamic fields on the longitudinal symmetry plane obtained from the simulation of pure copper printed at 800 W and 1000 mm/s. Convection-driven flow characterizes the gas fluid-dynamics reaching a maximum velocity intensity of 0.45 m/s. Slow convective flows are obtained in the melt pool due to the model simplifications.

**Figure 4:**
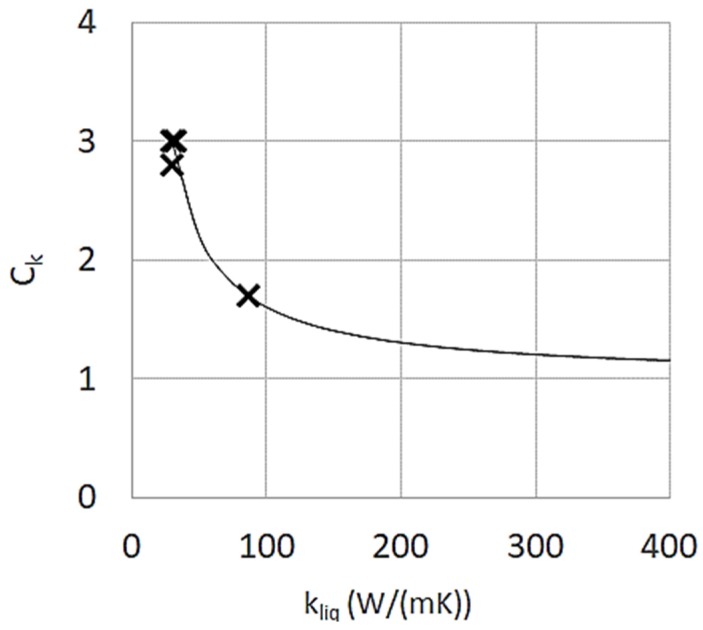
Fitted values of the multiplying parameter *C_k_*.

Track geometry data are available for four alloys: Ti6Al4V [30], Inconel625 [15], Al7050 [29], 316L [24]. For each alloy, the effective liquid thermal conductivity (*k_eff_*) has been derived running the model several times varying *k_eff_* until fitting the track geometry measured for all available sets of parameters. Assuming for the correlation between *k_eff_* and the actual liquid metal conductivity (*k_liq_*) the form:

(9) $$k_{eff}=C_{k}k_{liq}$$

from the *k_eff_* evaluated for each alloy, the corresponding values of *C_k_* have been calculated and plotted against *k_liq_* in Figure 4. The fitting trendline, has the following expression:

(10) $$C_{k}=1+\frac{60}{k_{liq}}$$

### 3.1. Comparison of Calculated and Measured Data of Track Geometry

**Figure 5:**
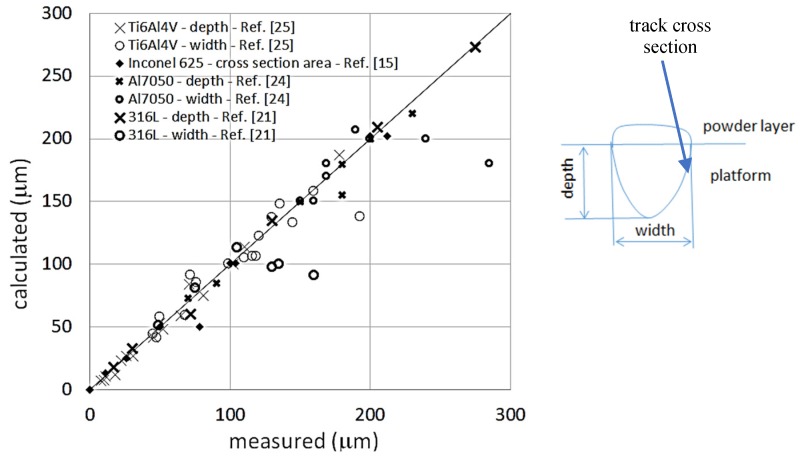
Comparison between measured and calculated data of track cross-section dimensions.

Comparison of resulting track dimensions with measured data is shown in Figure 5, in terms of cross section width and depth, according to the scheme shown in Figure 5 (alloys: Ti6Al4V, Al7050 and 316L) and cross section area (alloy Inconel 625).

An average good match has been found with exception of width values for deep keyhole shapes. The typical keyhole cross section geometry is characterized by a width profile rapidly changing from wide near the surface, to narrow deep below. The simplified approach of the present model is not capable of catching this geometry complexity. The calculated cross section area fits well with the area experimentally measured. Conversely, the calculated shape presents much uniform width all along the depth, predicting smaller width at the top of the track.

**Figure 6:**
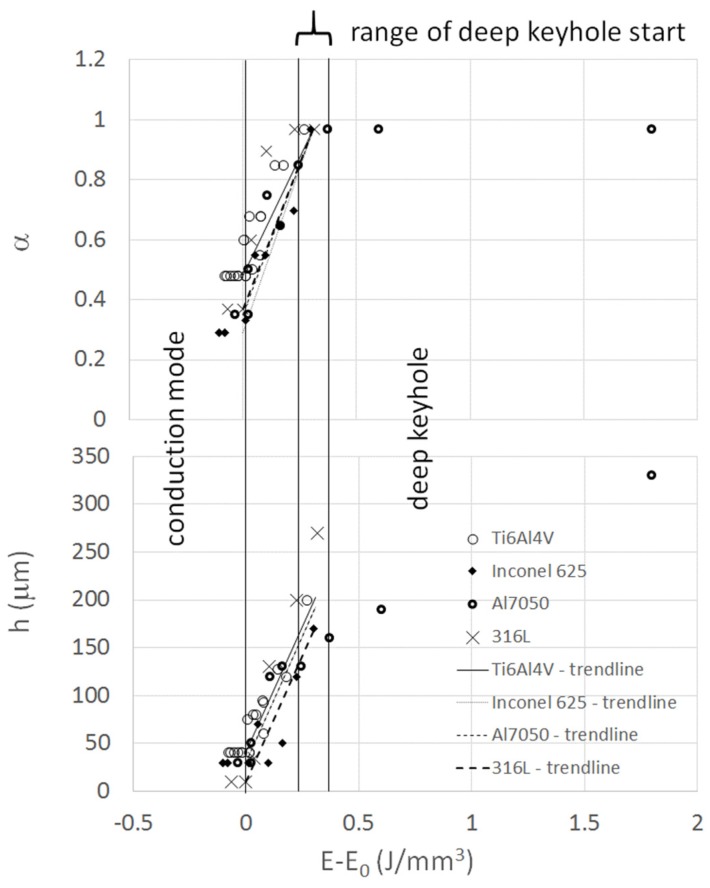
Values of absorptivity *α* and depth of energy deposition *h* vs the difference of specific energy and yield value of specific energy between conduction and evaporation. All adjusted values for the alloys: Ti6Al4V, Inconel 625, Al7050 and 316L are included in the diagram.

Corresponding adjusted values of *α* and *h* are found in Figure 6. The x axis of the diagram reports the difference between specific energy of the simulated track and the specific energy marking the threshold to boiling of the alloy (*E*_0_). The resulting rising trends of both variables within the transition range, are quite close for the four alloys. The straight lines superimposed in Figure 6 are trendlines used for the extrapolation of the model fitting parameters. They are derived through the following relationships:

(11) $$\alpha =\alpha_{0}+\frac{0.97-\alpha_{0}}{0.31}\left(E-E_{0}\right)$$

(12) $$\;h=h_{0}+\frac{163-h_{0}}{0.31}\;\left(E-E_{0}\right)$$

where the coefficients: 0.97, 0.31 and 163 must be regarded as adjusted parameters, which could be object of further refinement through calibration of other alloys. Deep keyhole start is defined as the specific energy difference (*E* − *E*_0_) at which the absorptivity reaches the maximum level. Figure 6 shows the range inside which this yield is met by all the analyzed alloys, denoted as range of deep keyhole start. 

The rising trend of *h* after deep keyhole formation (*α* = 0.97) has been measured for alloys Al7050 and 316L, showing different features. While the Al7050 track depth begins to increase less steeply after a deep keyhole appears, it keeps on following the same slope in 316L tracks. Maybe this could be ascribed to the reduced thickness of the 316L disks, used for laser scanning [24], becoming comparable to the track depth at high laser-specific energies.

Values of *α* resulting from the model calibration are directly comparable to measured ones derived through calorimetric measurements of absorbed energy for alloy 316L [24]. The absorptivity referred to in [24] represents the fraction of nominal energy being absorbed. Therefore, it corresponds to the product of laser absorptivity and efficiency (*α* × *η*), according to the scheme adopted in this work. Data referring to laser scans on bare disks have been used.

**Figure 7:**
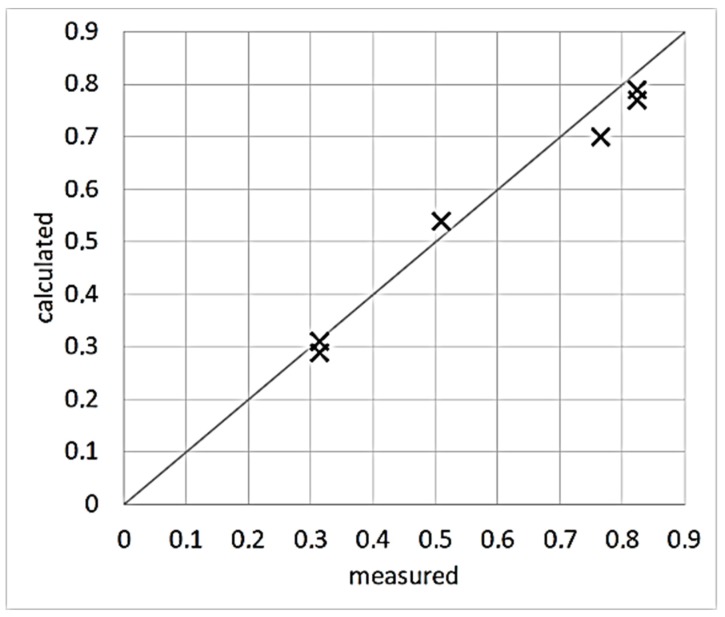
Comparison of measured [21] and calculated values of total absorptivity of alloy 316L.

Figure 7 shows the comparison of measured to calculated values of total absorptivity (*α* × *η*) of alloy 316L.

### 3.2. Comparison of Calculated and Measured Data of Relative Density

**Figure 8:**
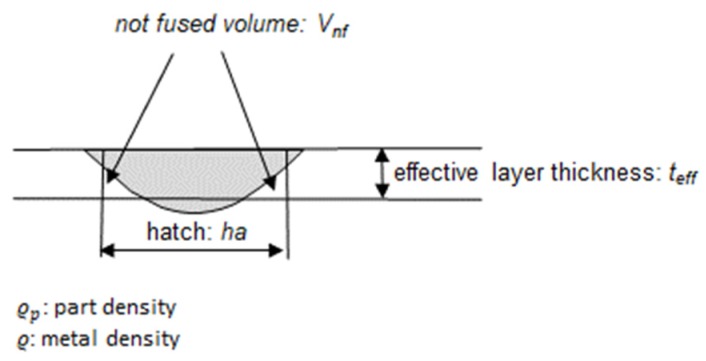
Scheme for the calculation of the part density from the calculated track geometry.

The part relative density is derived by simulating regime printing conditions for which the effective layer thickness: *t_eff_* is assumed, to be distinguished from the nominal thickness: *t_nom_*. The relationship between: *t_eff_* and *t_nom_* is approximated by: *t_eff_* = *t_nom_*/f [35], being f the metal fraction of the powder, assumed to be equal to 0.6 in this work. Figure 8 shows the scheme adopted for deriving relative density from the track geometry.

**Figure 9:**
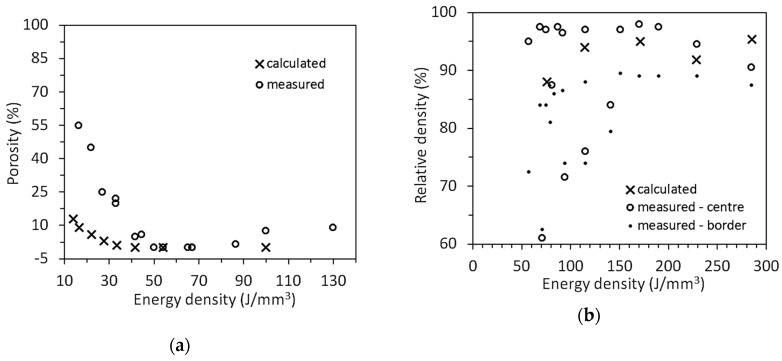
Comparison of measured and calculated values of: (**a**) porosity (Ti6Al4V, diagram taken from [30]); (**b**) relative density (pure copper, diagram taken from [31]).

Relative density has been calculated according to the scheme of Figure 8 for alloys Ti6Al4V and pure copper. Figure 9a,b show the calculated data of porosity (Ti6Al4V [30]) and of relative density (pure copper [31]) compared to measured data. Both diagrams present the energy density on the x axis expressed as:

(13) $$E=\frac{P}{v\;t_{nom}\;ha}$$

A discrepancy is visible among calculated and measured data in Figure 9a concerning:

1. porosity for *E* < 50 J/mm^3^ (porosity due to lack of fusion): calculated values are much lower than measured. Values of porosity as high as 65% are found in the reference source [30], which raise some doubt about the coherency of the measured sample. Therefore, no deep analysis about the reason of the discrepancy among calculated and measured data has been done, concerning the highest values, otherwise the information given by the measured porosity trend has been considered valid;
2. the calculated threshold of formation of porosity due to lack of fusion is calculated at 42 J/mm^3^ against the experimentally observed at 50 J/mm^3^;
3. the model cannot calculate porosity due to deep keyhole, but only the threshold which is found at *E* = 133 J/mm^3^. Experimental data show presence of 1% porosity at *E* = 87 J/mm^3^ and a net porosity increase above *E* = 100 J/mm^3^. Thus, keyhole porosity could already form for a calculated value of *α* equal to about 0.85.

Since no measured data of track geometry is found in [31], the simulation of pure copper beads has been performed using values of liquid pool thermal conductivity, *α* and *h*, derived from the calibration of the previously discussed four alloys. The resulting relative density fits rather well with experimental data, and thus no further adjustment has been added on pure copper. Figure 9b differentiates relative density measured in the central part of the specimens from the one measured in the borders. Calculated relative densities seem to be in quite good agreement with data relating to the specimen center.

## 4. Discussion

Although the analysed alloys have quite different physical properties, the track evolution with increasing input laser-specific energy is similar. Rising trends of *α* and *h* in Figure 6 show common features of cavity deepening in the transition mode for the four alloys. Each alloy has its specific surface absorptivity, so that pure copper absorbs much less energy than other alloys in the conduction mode. Each alloy also shows a different level of laser-specific energy above which the transition occurs. The model captures this energy level without resorting to experimental data. Increasing further laser specific energy results in reaching conditions of deep penetration and almost full energy absorption. This final condition is gained for an additional step of energy not depending on the alloy in a significant way. Starting from these considerations, the main difference in the printing behaviour of very different alloys seems to be mainly in the conduction mode features and in the threshold energy to the transition mode.

## 5. Conclusions

A model has been developed using the commercial code ANSYS Fluent for simulating the printing process inside a laser melting powder bed machine. 

The model has been built to meet the need to have a simple tool to design printing practices suitable for innovative alloys, not presently produced in the additive manufacturing route. For this reason, in this first step of development, available literature data concerning different alloys with different base metals have been collected and analyzed. Further model developments and applications to innovative alloys properly designed for the additive manufacturing production route are ongoing and will be the object of publication in the future. 

The model has been calibrated fitting experimental measures of track width, depth, cross sectional area, absorptivity and relative density taken from five literature sources, referring to: Ti6Al4V, Inconel 625, Al7050, 316L and pure copper.

A strategy of model calibration is employed based on varying the effective liquid pool thermal conductivity. The fitting strategy consists in obtaining the boiling temperature as maximum pool temperature at operating conditions for which the start of liquid pool boiling and cavity drilling are observed. Laser absorptivity and depth of application of laser energy are further varied in order to fit width and depth data. 

The development and adjustment of the model put into evidence the feature of the basic mechanism of the laser printing process, common to all alloys. It consists in the almost linear rise of laser absorptivity and depth of penetration with increasing specific energy. The assumed slopes result very close for the analyzed alloys, giving confidence about the use of the obtained trends for the prediction of the printing process of other alloys. In particular, laser absorptivity increases starting from the base level consistent with the absorptivity of the flat surface of the alloy at the laser wavelength. It finally reaches a maximum value close to unity. 

The model has been applied also to the prediction of the part density. This could be done only for pure copper and Ti6Al4V alloys for the availability of measures made at different laser operating conditions. Predicted porosity of parts is in qualitative agreement with experimental data for Ti6Al4V. Absolute values of calculated relative density are in good agreement with measured ones for pure copper. This very first result gives confidence on the suitability of the use of the model for the prediction of part density also for other alloys. As this is a key point of the model, further adjustments and refinements are ongoing.

## Figures and Tables

**Table 1:** Thermo-physical properties used in the model for the five commercial alloys used for the calibration. Experimental data have been found in the references shown in the table.

Property	Ti6Al4V	INC625	Al7050	316L	Cu(99,9%)
Density (kg/m^3^)	4000	8440	2810	7890	8960
Liquidus Temperature: T_liq_ (K)	1986	1607	906	1710	1356
Solidus Temperature: T_sol_ (K)	1970	1513	787	1608	1356
Specific Heat (J/(kgK))					
T_amb_	550	440	860	450	481
T_sol_	830	650	1050	750	481
T_liq_	980	670	1120	770	531
Thermal Conductivity (W/(mK))					
T_amb_	5	11	117	16	397
T_sol_	32	30	156	33	317
T_liq_	32	30	87	30	157
Latent Heat of Fusion (J/m^3^)·10^9^	1.4	1.99	1.05	1.37	2.07
Boiling Temperature (K)	3600	3000	2800	3100	2840
Reflectivity at 1.06 μm (T≈900K)	0.52	0.71	0.65	0.63	0.84
Reference	[30]	[15]	[29]	[24]	[31]

## Nomenclature

*C_k_*	multiplying factor
*D*	distribution factor
*f*	metal fraction in the powder bed
*f_l_*	metal liquid fraction in the mushy zone
*g_i_*	gravity acceleration components (m s^−2^)
*h*	height of the cylinder of laser heat application
*ha*	hatch spacing (mm)
*H*	enthalpy (J kg^−1^)
*k_eff_*	effective thermal conductivity (W m^−1^ K^−1^)
*k_liq_*	thermal conductivity of the liquid metal (W m^−1^ K^−1^)
*K*	mushy zone permeability (m^2^)
*p*	pressure (Pa)
*P*	laser power (W)
*PDAS*	primary dendrite arm spacing (m)
*r_l_*	laser beam radius
*S_v_*	volumetric heat source (J m^3^)
*t*	time (s)
*t_eff_*	printing regime bed powder thickness (μm)
*t_nom_*	nominal powder bed thickness (μm)
*T_liq_*	liquidus temperature (K)
*T_sol_*	solidus temperature (K)
*u_i_*, *u_j_*	velocity components (m s^−1^)
*v*	scanning speed (m s^−1^)
*x*, *y*	coordinates in a frame relative to the laser beam (m)
*x_i_*, *x_j_*	coordinates (m)
*α*	laser absorptivity
*β*	fraction of the track surface contacting powder
*η*	laser efficiency
*μ*	viscosity (Pa s)
*ρ*	metal density (kg m^−3^)
*ρ_p_*	part density (kg m^−3^)
